# Cross‐Cultural Similarities and Differences in the Development of Infants' Positive Reactivity

**DOI:** 10.1111/infa.70039

**Published:** 2025-08-22

**Authors:** Helen Wefers, Nils Schuhmacher, Joscha Kärtner

**Affiliations:** ^1^ Department of Psychology University of Münster Münster Germany

**Keywords:** culture, developmental trajectories, early infancy, face‐to‐face interaction, positive affect

## Abstract

To investigate the development of positive affect during early infancy across cultures, we conducted a joyful affect–eliciting dyadic face‐to‐face interaction between a female experimenter and 3‐ and 4.5‐month‐old infants from Münster (urban Germany; *n* = 20 at 3 months, *n* = 20 at 4.5 months) and indigenous Kichwa families from the Andean context (rural Ecuador; *n* = 24 at 3 months, *n* = 27 at 4.5 months), which differ in their ethnotheories about infants' ideal affect. Results pointed to cross‐cultural differences in infants' affective reactivity to high‐intensity stimulation, namely higher intensities of positive affect at 3 months in Münster as compared to Kichwa infants that disappeared at 4.5 months of age. The findings serve as an important complement to naturalistic studies that have left open the question of the developmental continuity of cross‐cultural differences in infant positive affect beyond 3 months. We discuss our findings in terms of a dynamic interaction between culturally informed parent‐infant interactions and biological potentials that give rise to both cross‐cultural similarities *and* differences in the course of emotional development, even in early infancy.

## 1 Introduction

Previous naturalistic studies have suggested that during the sensitive phase around one's second month of life, positive affect, which comprises positively valanced emotions, is socialized differently across cultures (LeVine et al. 1994; Wörmann et al. 2012, 2014). A central assumption in this context is that culturally embedded and culture‐specific ethnotheories, the shared and abstract mental schemas that organize parental behavior (Harkness et al. 2007), function as a guiding factor regarding parental emotion socialization in infancy. It follows that infants gain culture‐specific interactional experiences, which are in turn internalized (Holodynski and Friedlmeier 2006; Sameroff 2010; Stern 2018), and—on these grounds—lead infants to display culture‐specific affective reactions during everyday interactions with their caregivers (LeVine et al. 1994; Wörmann et al. 2012, 2014). Because earlier cross‐cultural studies on infants' emotional development have mostly relied on naturalistic mother‐infant interaction, it remains untested whether infants from different cultures respond with different levels of positive affect to social stimuli intended to create standardized target states of the social interaction. To investigate this research question in two cultural milieus, which differ in their ethnotheories about infants' ideal affect, we conducted standardized positive affect–eliciting distal face‐to‐face interactions with 3‐ and 4.5‐month‐old infants from Münster (urban Germany) and indigenous Kichwa families from the Andean context (rural Ecuador).

Regarding early emotional development, neonates initially express unfocused expression signs (i.e., crying) and body reactions (physiological and psycho‐endocrinological reactions), so‐called precursor emotions (Sroufe 2009); *precursor*, because elementary components of fully functioning emotion systems are initially lacking or underdeveloped. That is, in line with the internalization model of emotional development (Holodynski and Friedlmeier 2006), we define emotions as a functional psychological system that involves the interplay of several components (i.e., situational appraisals, action readiness and expression, bodily changes, and subjective feelings; Holodynski and Seeger 2019). Those precursor emotions become more differentiated over time as those signals are mirrored, co‐regulated and co‐constructed by caregivers during everyday interactions (Holodynski and Friedlmeier 2006; Holodynski and Seeger 2019). At the biological level, a key developmental transformation is the so‐called 2‐month shift, which provides the developmental potentiality for infants to actively share emotions within face‐to‐face communication (Kärtner et al. 2010; Wörmann et al. 2012, 2014).

Lavelli and Fogel (2002, 2005) investigated the development of positive affect within mother‐infant dyads from urban Italy and identified that—at the age of 8 weeks—infants begin to actively engage in face‐to‐face communication (from simple gazing to showing facial actions such as brow raising, mouth opening, and smiles). Mothers in turn mark and amplify those communicative infant actions such that maternal talking and smiling, infant smiling, and infant cooing are bidirectionally linked and cycle between each other, suggesting the existence of a positive emotional attractor in their social communication system. Looking more closely at the contribution of specific parenting behaviors to infants' social expressiveness, specific *forms* of (contingent) maternal responses—that is mirroring and marking with a smile—are critical for the increase in these infant behaviors over time (Murray et al. 2016).

From a culture‐sensitive viewpoint, caregivers' perception and reaction to their infants' affective signals are culturally shaped (LeVine et al. 1994; Wörmann et al. 2012, 2014). For example, the likelihood for infants to receive affect‐mirroring contingent responses differs between cultures and varies with the valence of infants' affective signals (Broesch et al. 2016).

Accordingly, initial empirical evidence has pointed out cross‐cultural differences in infants' affect‐expressive behavior: Analyzing the emergence of social smiling in naturalistic mother‐infant interactions, previous cross‐cultural studies have reported culture‐specific affect displays appearing around the 2‐month shift (Kärtner et al. 2022; Keller and Kärtner 2013; Wörmann et al. 2012, 2014). Specifically, an increase in the duration of infant smiling (i.e., between postnatal weeks 6 and 8; Wörmann et al. 2012, 2014) and an increase in infants' high‐intensity positive affect (i.e., from 9 to 13 weeks; Kärtner et al. 2022) was found only in the sample from urban Germany, a cultural milieu that is associated with a preference for high levels of positive affectivity (Wefers et al. 2022), but not in samples from rural Cameroon and rural Ecuador. From these findings, Wefers et al. (2022) and Wörmann et al. (2012, 2014) argued that cultural differences in infant smiling develop within culturally informed interactions with caregivers, and they discussed culture‐specific ethnotheories as a guiding factor regarding parental emotion socialization in infancy (see also Kärtner 2015; Keller and Kärtner 2013). However, those initial findings on infants' affective reactions are all based on naturalistic designs and, thus, on a combination of infants' behavioral inclinations and potentially culture‐specific social inputs of caregivers.

A complementary methodological approach to analyzing cultural specificities in the development of positive affect, which allows to draw conclusions about previous interaction experiences and their developmental effectiveness, is to assess infants' reactions to a *standardized* social input. Moreover, previous studies (Kärtner et al. 2022; Wörmann et al. 2012, 2014) focused on the developmental period shortly before, during and after the 2‐month shift, leaving open the question of the developmental continuity of cross‐cultural differences in infant positive affect beyond 3 months. More generally, we argue that—to advance our understanding of emotional development during infancy—it is important to study the development of positive affect in infants whose interaction experiences with caregivers—such as interactional routines around positive affect—differ systematically, such as between cultures.

Against this background, we conducted standardized, joyful affect–eliciting dyadic face‐to‐face interactions between an experimenter (native female strangers) and infants from the same overall project as Wefers et al. (2022, 2023), namely from the two cultural milieus Münster (urban Germany) and the Kichwa ethnic group from the northern Andes region (rural Ecuador), as these milieus have been reported to differ regarding their preferences for high‐ versus low‐arousal positive affect (Wefers et al. 2022). As we were interested in the developmental trajectories of infants' positive affect display in reaction to positive stimulation (i.e., infants' *positive reactivity*) beyond the 2‐month shift, we tested infants' reactions at 3 months, when—biologically—infants' potential for face‐to‐face interaction is very high, and at 4.5 months, when infants begin to shift their attention from human faces to objects (Nomikou et al. 2016).

### 1.1 Hypotheses on Infants' Positive Reactivity and Focus of Attention at 3 and 4.5 Months

In order to investigate cross‐cultural similarities and differences in the development of positive affect in infants and to explore the developmental trajectory of this facet of emotional expressivity, we standardized a specific format of social interaction, namely positive affect‐eliciting distal face‐to‐face interactions, and conducted those standardized assessments with Kichwa infants and infants from Münster at 3 and 4.5 months of age. More precisely, the amount of stimulation of positive affectivity offered by the experimenter, who elicited and mirrored infant smiles, increased in the course of the standardized interaction.

This specific format of social interaction—that is, its distal format and especially the experience of high‐intensity positive affect—was assumed to be closer to the average expectable environment (LeVine 1990) of infants from Münster, who experience more distal parenting, than of Kichwa infants, who experience more bodily proximity during everyday routines and presumably experience low‐arousal positive affect when distal modes of interaction are established (Wefers et al. 2022).

Based on infants' culture‐specific internalized repertoire of interaction experiences (Holodynski and Friedlmeier 2006; Sameroff 2010; i.e., socialization toward high‐ vs. low‐intensity positive affect), infants were expected to react differently to this specific interactional style; more precisely, the closer the interactional style is to their everyday experience, the more strongly they should engage in those patterns of mutual engagement. As a consequence, we expected infants from Münster (urban Germany) to display higher intensities of positive affect as compared to Kichwa infants, especially during the high‐intensity stimulation phase of the experiment, when the interactional style is even closer to the ideal of caregivers from Münster, who—according to a recent study based on the same samples (Wefers et al. 2022)—have a stronger preference for high‐intensity positive affect as compared to primary caregivers from the Kichwa ethnic group from the northern Andes region (rural Ecuador).

Since most cross‐cultural studies have addressed similarities and differences in infant positive affect at 3 months of age, this hypothesis refers to 3‐month‐olds. Due to the paucity of behavioral and observational cross‐cultural studies on developmental trajectories of positive affect (or of other emotions) beyond 3 months of age, we do not have a specific hypothesis regarding infants' affective reactions at 4.5 months of age, but we aim to explore the question: Does the expected gap in positive affectivity at 3 months of age persist or widen due to accumulating experience as conceptualized in developmental cascade models (Masten and Cicchetti 2010), or do those cross‐cultural differences in infant affect disappear, for example, because Kichwa infants develop this type of positive reactivity *after* the third months? In parallel with infant affect, we explored infants' attention to faces of social interaction partners at 3 and 4.5 months in order to see whether possible intercultural differences in affect go along with culture‐specific gaze patterns.

## 2 Methods

### 2.1 General Procedures

The present study was part of a larger cross‐cultural longitudinal project that focused on early social expectations (Wefers et al. 2023), maternal ethnotheories about infant affect and activity (Wefers et al. 2022) and mother‐infant interaction around infant smiling (Kärtner et al. 2022). Data assessments of the overall project took place from 2017 to 2018 with weekly home visits in postnatal weeks 7–18. The participating families were informed about the data protection policy and signed an informed consent before the first data assessment. The standardized positive stimulation by an experimenter (native female stranger) was implemented in weeks 12 and 18. The experimenter (same research assistant for all families in Ecuador, six research assistants in Germany) was the same at 12 and 18 weeks. For the main data collection of the present study in Ecuador, we selected mothers and infants who identified themselves as Kichwas, who lived in communities in the larger surroundings of Cotacachi or Otavalo and who gave birth no more than 6 weeks prior to the start of the study. The hospitals of Cotacachi and Otavalo provided us with information about newborn children. A local research assistant visited the families, informed them about the study, and invited them to participate.

In Germany, we included mothers and infants who lived in the city of Münster and who recently gave birth. We contacted families by post after receiving their contact information from the local registration office or invited them personally during prenatal classes.

### 2.2 Assessing Infants' Positive Reactivity

When infants were 3 and 4.5 months old, two local research assistants visited the families at their homes to conduct a modified still‐face procedure. For the purpose of the present study, we analyzed infants' positive reactivity during the *modified two‐phase baseline* of the still‐face procedure^1^: During the two phases of the assessment, the experimenter engaged in face‐to‐face interaction, contingently mirroring the infants' facial expressions and encouraging infant smiles, while systematically increasing the amount of stimulation from one phase to the other. Specifically, the intensity of the experimenter's smiles increased from medium‐intensity smiles (stimulation Phase 1) to high‐intensity smiles (stimulation Phase 2). That is, we standardized the *target states* of the social interactions and the experimenters were instructed to stimulate the according target states during Phase 1 (medium‐intensity) and 2 (high‐intensity). We instructed the experimenter to refrain from body contact during both phases. The use of language was adapted to the degree of stimulation. These aspects of the experimenter's interaction behavior were checked during the piloting phase.

During the assessments at both ages, infants were placed in a child seat, and the experimenter sat in front of the seat with no toys, facing the child. A GoPro Hero camera was installed on the backrest of the child seat, filming the experimenter, and a Panasonic HC Camcorder camera was placed on a tripod, filming the infant. The second research assistant gave a signal when the next phase of the experiment started.

### 2.3 Participants

We were interested in meaningful effects (i.e., medium to large effect sizes with *f* ^2^ > 0.30) and calculated sample size based on a corresponding power analysis with GPower for each mixed analysis of variance (culture × phase) at both 3 and 4.5 months of age (i.e., test family: *F* tests; statistical test: ANOVA, repeated measures, within‐between interaction), based on the following input parameters: two tails, *f* ^2^ = 0.30, *α* = 0.05, power (= 1 − *β*) = 0.80, number of groups = 2, number of measurements = 2, correlation among repeated measures = 0.5, nonsphericity correction *ε* = 1. The result indicated that a total sample size of *N* = 24 would be sufficient to detect corresponding effects. For a longitudinal mixed ANOVA (culture × age × phase), including age as a further factor, the total sample size would increase to *N* = 28.

All details of the ethical guidelines have been followed. This research was conducted in accordance with the Declaration of Helsinki and the Ethical Principles of the German Psychological Society, the Association of German Professional Psychologists, and the American Psychological Association (APA). It involved no invasive or otherwise ethically problematic techniques and no deception (and therefore, according to National jurisdiction, does not require a separate vote by a local Institutional Review Board; see the regulations on freedom of research in the German Constitution (§ 5 (3)), and the German University Law (§ 22)). In Ecuador, we conducted the study in cooperation with the University of Otavalo and the Union of Farmer and Indigenous Organizations of Cotacachi (UNORCAC). The study was approved by the scientific commission of the University of Otavalo.

A total of 27 families from urban Germany and 29 families from rural Ecuador participated in the study, resulting in *n* = 56 potential sessions at 3 months and also at 4.5 months. Given the focus of our research question, we included all assessments in which infants successfully completed both phases of the standardized assessments (stimulation Phase 1: medium‐intensity stimulation; stimulation Phase 2: high‐intensity stimulation). From the 112 potential sessions, 110 sessions were realized since one session at 3 months and one session at 4.5 months from Germany did not take place, either because of regulatory difficulties associated with the infant (excessive crying, *n* = 1) or because a family went on a holiday for several weeks (*n* = 1). Furthermore, some sessions were not completed or had to be excluded for different reasons, such as fussiness or whining (*n* = 19; see Appendix A for details), resulting in a final set of *N* = 91 standardized assessments (3 months: *n*_MS_ = 20, *n*_KI_ = 24; 4.5 months: *n*_MS_ = 20, *n*_KI_ = 27).

### 2.4 Demographics and Description of Cultural Milieus

Information about the two cultural milieus is presented in more detail in Table 1. Samples significantly differed in household sizes and parents' formal education but did not differ significantly with regard to parity and infant gender. Mothers who had migrated to Münster from abroad were also included (see Table 1): Four mothers living in Münster had migrated—two as children and two as adults—from Kazakhstan, Sri Lanka, Russia, or Hungary, whereas none of the Kichwa mothers had. We asked mothers where their youngest child is located while they follow their daily routines, and we categorized their free responses (mainly distant; mainly in body contact; both positions equally). As shown in Table 1, infants in Münster were mainly at a distance to their mothers (84.0%), while for Kichwa infants all categories occurred equally often (mainly distant = 31.0%, mainly in body contact = 34.5%, both positions equally = 34.5%; for a more detailed description of the families' cultural niches, see Wefers et al. 2022).

**TABLE 1:** Demographic information and description of cultural milieus.

Sociodemographic variable		Cultural milieu		Statistical significance
		Münster	Kichwa	
		% or *M* (SD)	% or *M* (SD)	
Gender (% girls)		40.0%	62.1%	*χ* ^2^ = 2.62, *ɸ* = −0.22
Parity (% firstborn)		52.0%	34.5%	*χ* ^2^ = 1.69, *ɸ* = 0.18
Age mothers (in years)		33.48 (3.82)	28.69 (7.37)	*t* = −3.06**, *d* = 0.82
Age fathers (in years)		36.96 (5.91)	32.90 (8.04)	*t* = −1.98*, *d* = 0.60
Migratory experience mothers		*N* = 4	*N* = 0	*p* (Fisher) = 0.040*, ɸ =* 0.31
Time since mothers migrated (in years)		Mdn = 15.00	—	
		MIN = 4.00		
		MAX = 32.00		
Infant's location during everyday routines	Mainly distant	84.0%	31.0%	*χ* ^2^(2) = 15.25**, *ɸ* = 0.53
	Mainly proximal	8.0%	34.5%	
	Both positions equally	8.0%	34.5%	
Mothers' partnership status	Married	76.0%	69.0%	*p* (Fisher) *=* 0.192, *C* = 0.26
	Living with partner	24.0%	17.2%	
	Single partners	0.0%	13.8%	
Household sizes		3.64 (0.91)	7.28 (2.85)	*t* = 6.49**, *d* = 1.72
Number of siblings		0.64 (0.91)	1.62 (2.03)	*t* = 2.35*, *d* = 0.62
Formal education, mothers (in years)		15.32 (2.97)	8.55 (3.95)	*t* = −7.02**, *d* = 1.94
Formal education, fathers (in years)		15.88 (3.42)	7.56 (4.28)	*t* = −7.59, *d* = 2.15
Acquisition of a profession^a^, mothers		Yes: 100%	Yes: 58.6%	*χ* ^2^ = 13.30**, *ɸ* = 0.50
		No: —	No: 41.4%	

^a^ Professions including formally acquired and self‐learned skills.

*p < 0.05, **p < 0.01; two‐tailed.

### 2.5 Behavioral Coding and Reliabilities

Based on an interval coding approach with 1‐s intervals, affective reactions as expressed by facial expressions and vocalizations were coded separately during Phase 1 and 2, using Mangold Interact (version 16.1.5.8). We opted for this multimodal approach in line with others who focused on facial and vocal emotional expressions during early communication with the mother (e.g., Lavelli and Fogel 2005). To gain information about the infants' focus of attention at 3 and 4.5 months of age, infant gaze was coded. The average length of the phases was as follows: Phase 1 = 60 s (SD = 5 s), Phase 2 = 62 s (SD = 2 s). While there were no significant cultural differences at 4.5 months, the phases 1 and 2 were slightly longer in the Kichwa milieu at 3 months of age: Phase 1 at 3 months: *M*_MS_ = 58 s, SD_MS_ = 6 s; *M*_KI_ = 62 s, SD_KI_ = 5 s; *t*(42) = 2.16, *p* = 0.037; Phase 2 at 3 months: *M*_MS_ = 60 s, SD_MS_ = 2 s; *M*_KI_ = 63 s, SD_KI_ = 3 s; *t*(39.48) = 4.09, *p* < 0.001.

#### 2.5.1 Facial Expressions

Each second was coded as either neutral facial expression, negative affect (aversive responses: brow knitting, lower lip raising, horizontal stretching of lip corners), positive affect (infant smile) or not codable. Infant smile was further coded for intensity of positive affect on a 4‐point scale based on the combinations of smiling lip corner movement (AU12), eye constriction (AU6) due to the cheeks being raised, and mouth opening (AU25) with intensities ranging from very low, low, middle to high (Ekman and Friesen 1978; see Table 2 for coding criteria for the intensities of infant smiling).

**TABLE 2:** Description of coding criteria for the intensities of infant smiling.

Smiling intensity	Coding criteria
Very low (1)	Slight raising or sideways movement of lip corners without eye constriction, cheeks not raised, mouth closed
Low (2)	Mild raising or sideways movement of lip corners without eye constriction, cheeks slightly raised, mouth can be open
Middle (3)	Distinct raising or sideways movement of lip corners with eye constriction, cheeks clearly raised, mouth can be open
High (4)	Open mouth that is intensely smiling (i.e., intense raising or sideways movement of lip corners) or laughing, strong eye constriction, and highly raised cheeks

*Note:* The numeric value assigned to each level of smiling is provided in parenthesis.

#### 2.5.2 Vocalizations

Each second was coded as either no vocalization, neutral vocalization (containing neither a positive nor a negative valence), negative vocalization (whining, whimpering, crying, but also angry and sad vocalizations), positive vocalization, vocalization could not be coded (e.g., not audible due to background noise) or *other* sounds (e.g., unvoiced sounds, vegetative sounds such as hiccups). Positive vocalizations were further coded for intensity of positive affect on a 3‐point scale with intensities ranging from low, middle to high (see Table 3 for coding criteria for positive vocalizations).

**TABLE 3:** Description of coding criteria for the intensities of positively valanced vocalizations.

Intensity of positive vocalization	Coding criteria
Low (1)	Cooing, low‐intensity sounds of contentment
Middle (2)	Mild laughter, sounds of contentment with very positive valence
High (3)	Open laughter

*Note:* The numeric value assigned to each level of positive vocalizations is provided in parenthesis.

#### 2.5.3 Gaze

We used the following categories to code infant attentive reactions: gazing at the experimenter (gaze is focused on her face during the complete 1‐s interval), not gazing at the experimenter (not focused on her face, eyes opened), switching gaze (between focusing on the experimenter's face and not focusing), eyes closed, and gazing could not be coded (e.g., due to hidden face).

#### 2.5.4 Reliabilities

We calculated interrater reliabilities between a gold standard (doctoral student) and one coder (trained German research student) in the case of vocalizations and between the same gold standard and two independent coders (also trained German research students) in the cases of facial expressions and gaze for 11% of the 91 videotaped assessments, resulting in approx. 1290 independently coded 1‐s intervals. Within this set, cultural milieus and measurement points were equally distributed. Reliabilities were computed after excluding non‐codable intervals. For vocalizations, we computed Cohen's kappa, which resulted in *κ* = 0.70, indicating a substantial strength of agreement (Landis and Koch 1977). For facial expressions, we computed a weighted Cohen's kappa (quadratic weights) for ordinal data, and all *κ*s exceeded 0.72, indicating high reliability. For infant gaze, we also computed Cohen's kappa, and all *κ*s exceeded 0.75, indicating high reliability. After calculating reliabilities, vocalizations were coded by one coder and the coding of facial expressions and gaze was realized by two independent coders.

### 2.6 Dependent Variable for Intensity of Infant Positive Affect and Focus of Attention

Based on the 1‐s codings of infant facial expressions and vocalizations, we integrated both modalities in the following way to compute *an intensity of positive affect* score from all codable 1‐s intervals per phase (i.e., Phase 1 and 2): Given that high‐intensity smile was only coded in 0.40% of the coded infants' smiles, we (i) recoded high‐intensity into middle‐intensity smiles, resulting in three levels of intensity of infant smile, (ii) summed up the intensity of social smile with the intensity of positive vocalizations to form a positive affect intensity score per 1‐s interval (scales ranging between 0 and 6 for each interval; e.g., with middle‐ to high‐intensity smile in combination with open laughter scored as 6), and (iii) finally computed average intensities of positive affect from all codable 1‐s intervals for each phase.

Means and standard deviations for the final intensity of positive affect as well as for the intensities of positive affect separately for each modality (as expressed by facial expressions and by vocalizations) during Phase 1 and 2 at both ages are presented in Table 4. On average, intensities of positive affect were relatively low (see Table 4).

**TABLE 4:** Means and standard deviations for intensity of positive affect during Phase 1 and 2 at 3 and 4.5 months.

	Phase 1		Phase 2	
	Münster	Kichwa	Münster	Kichwa
	*M* (SD)	*M* (SD)	*M* (SD)	*M* (SD)
3 months				
Multimodal score				
Positive affect [0–6]	0.57 (0.57)	0.60 (0.55)	0.67 (0.54)	0.39 (0.32)
Single‐modality scores				
Smiling [0–3]	0.56 (0.56)	0.60 (0.54)	0.63 (0.54)	0.38 (0.32)
Positive vocalizations [0–3]	0.02 (0.03)	0.01 (0.02)	0.03 (0.05)	0.01 (0.03)
4.5 months				
Multimodal score				
Positive affect [0–6]	0.38 (0.44)	0.32 (0.33)	0.55 (0.48)	0.55 (0.63)
Single‐modality scores				
Smiling [0–3]	0.37 (0.43)	0.32 (0.33)	0.50 (0.45)	0.54 (0.63)
Positive vocalizations [0–3]	0.01 (0.01)	0.00 (0.01)	0.05 (0.05)	0.02 (0.04)

*Note:* Positive reactivity at 3 months: *N* = 20 for the Münster sample and *N* = 24 for the Kichwa sample. At 4.5 months: *N* = 20 for the Münster sample and *N* = 27 for the Kichwa sample. In most cases (96.0% of intervals with positive affect), positive affect was expressed in only one modality (i.e., 94.9% with only smiling, and 1.1% with only vocal expression), while there was a bimodal expression in 4.0% of intervals.

*Infant gaze* was computed as the relative frequency of the code gaze at the experimenter, that is, number of intervals with infant gaze at the experimenter divided by all codable intervals per phase (i.e., Phase 1 and 2).

Importantly, in most cases (i.e., 90.6%) positive affect occurred during face‐to‐face interaction (i.e., either gazing at experimenter or switching gaze).

### 2.7 Plan of Analysis

We used SPSS (version 26) for the analysis of infants' affective reactions and focus of attention during both phases of the experiment. Due to the pattern of missing data (i.e., single assessments missing at one of the two ages) and in order to maintain a greater statistical power, for the main analyses, we computed separate ANOVAs for each measurement point (at 3 and 4.5 months). More precisely, we conducted mixed ANOVAs with *intensity of positive affect* and *infant gaze* as dependent variables, phase (Phase 1 vs. Phase 2) as the within‐subject factor, and infants' cultural milieu (Münster vs. Kichwa) as the between‐subject factor. We consider those effects as meaningful that are statistically significant (*p* < 0.05), but do also consider findings with medium effect sizes (i.e., *η* ^2^ ≥ 0.06, |*d*| ≥ 0.5). In case of significant interactions and interactions with medium effect sizes (i.e., *η* ^2^ ≥ 0.06), we conducted post‐hoc *t*‐tests following the recommendations by Jaccard and Turrisi (2003).

Preliminary analyses had shown that neither the main effects of gender and parity nor their interactions with culture and phase were significant when included as additional factors in our ANOVAs, with only few exceptions (see Appendix B). We therefore decided to drop them from the final analyses.

As a complement to the main analyses, we conducted longitudinal analyses of infants' affective reactivity and gaze (*n* = 16 for the Münster and *n* = 22 for the Kichwa sample). To explore whether the effects of culture and phase change with infants' age, we conducted three‐factorial ANOVAs based on a reduced sample of infants for which longitudinal data were available with intensity of positive affect and infant gaze as dependent variables, age (3 vs. 4.5 months) and phase (Phase 1 vs. Phase 2) as within‐subject factors, and infants' cultural milieu (Münster vs. Kichwa) as the between‐subject factor. In case of significant three‐way age × phase × cultural milieu interactions, we conducted two‐factorial ANOVAs and post‐hoc *t*‐tests separately for each cultural milieu.

In case the cross‐sectional or longitudinal analyses of infants' affective reactivity or infant gaze yielded significant effects, we conducted binomial tests to test whether the effect could be confirmed for a significant proportion of infants. As a further complement, we additionally conducted cross‐sectional and longitudinal analyses of the (relative) occurrence of positive affective expressions.

## 3 Results

### 3.1 Positive Reactivity at 3 Months

**FIGURE 1:**
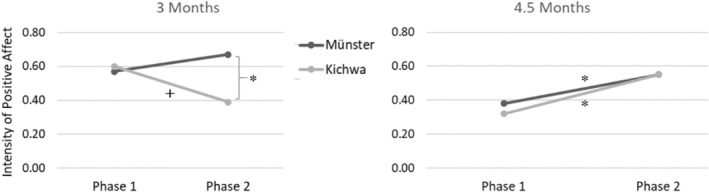
Average intensity of positive affect during Phase 1 and 2 at 3 and 4.5 months. ^+^ *p* < 0.10, *p < 0.05.

Regarding the intensity of positive affect at 3 months, neither the effect of phase *F*(1, 42) = 0.60, *p* = 0.445, *η* ^2^ = 0.01, nor the effect of culture was significant, *F*(1, 42) = 0.93, *p* = 0.340, *η* ^2^ = 0.02. However, there was a non‐significant cultural milieu × phase interaction with a medium effect size, *F*(1, 42) = 3.84, *p* = 0.057, *η* ^2^ = 0.08, indicating that changes in infant affect from Phase 1 to Phase 2 varied across cultures. More specifically, post‐hoc *t*‐tests yielded a non‐significant, descriptive decrease of infant positive affect from Phase 1 to Phase 2 in the Kichwa sample with a small to medium effect size, *t*(23) = 2.03, *p* = 0.054, *d* = 0.41. On an individual level, there was a decrease in 15 out of 24 Kichwa infants, *p* = 0.307. Münster infants showed a descriptive increase in positive affect across phases that was insignificant, *t*(19) = −0.80, *p* = 0.432, *d* = 0.18. Comparing the two cultures directly, post‐hoc *t*‐tests indicated that there was no difference between samples in the intensity of positive affect during Phase 1, *t*(42) = 0.17, *p* = 0.863, *d* = 0.05, but that the positive affect of infants from Münster was significantly more intense during Phase 2, *t*(42) = −2.13, *p* = 0.039, *d* = 0.65 (see Table 4 and Figure 1).

### 3.2 Positive Reactivity at 4.5 Months

With respect to the intensity of positive affect at 4.5 months, the effect of phase was significant *F*(1, 45) = 8.19, *p* = 0.006, *η* ^2^ = 0.15, indicating an increase in positive affect from Phase 1 to Phase 2 across cultures. On an individual level, there was an increase in 31 out of 47 infants, *p* = 0.040. At the same time, neither the effect of culture, *F*(1, 45) = 0.04, *p* = 0.850, *η* ^2^ = 0.00, nor the cultural milieu × phase interaction was significant, *F*(1, 45) = 0.21, *p* = 0.652, *η* ^2^ = 0.01 (see Table 4 and Figure 1).

### 3.3 Longitudinal Analysis of Infants' Positive Reactivity Across Ages

Regarding the intensity of positive affect, there were no significant main effects of age *F*(1, 36) = 1.43, *p* = 0.240, *η* ^2^ = 0.04, phase *F*(1, 36) = 1.54, *p* = 0.222, *η* ^2^ = 0.04, or cultural milieu, *F*(1, 36) = 0.01, *p* = 0.909, *η* ^2^ = 0.00. Neither the age × cultural milieu nor the phase × cultural milieu interaction was significant, dfs < 0.97, *p*s > 0.33, *η* ^2^s < 0.03. Furthermore, there was a non‐significant age × phase interaction with a medium to large effect size, *F*(1, 36) = 3.81, *p* = 0.059, *η* ^2^ = 0.10, and a significant three‐way interaction, *F*(1, 74) = 4.77, *p* = 0.036, *η* ^2^ = 0.12. This age × phase × cultural milieu interaction indicates that the age‐related change in infants' positive reactivity to different phases of positive stimulation varied with infants' cultural milieu.

To analyze this further, we conducted post‐hoc ANOVAs separately for each cultural milieu with age (3 vs. 4.5 months) and phase (1 vs. 2) as within‐subject factors. In the Münster sample, there were no significant main or interaction effects, *F*s < 1.58*, p*s > 0.22, *η* ^2^s < 0.10. For Kichwa infants, there were no significant main effects of age, *F*(1, 21) = 0.06, *p =* 0.809, *η* ^2^ < 0.00, or phase, *F*(1, 21) = 0.05*, p* = 0.822, *η* ^2^ < 0.00. However, there was a significant age × phase interaction, *F*(1, 21) = 7.87*, p* = 0.011, *η* ^2^ = 0.27. More precisely, according to post‐hoc *t*‐tests and in line with the results of the main analyses reported above, whereas 3‐month‐old Kichwa infants showed a non‐significant, descriptive *decrease* in positive affect from Phase 1 (*M* = 0.65, SD = 0.55) to Phase 2 (*M* = 0.42, SD = 0.32) with a small to medium effect size, *t*(21) = 2.02, *p* = 0.057, *d* = 0.43, with individual decreases in 14 out of 22 infants, *p* = 0.286, 4.5‐month‐old Kichwa infants significantly *increased* in positive affect from Phase 1 (*M* = 0.38, SD = 0.34) to Phase 2 (*M* = 0.64, SD = 0.66), *t*(21) = −2.35, *p* = 0.028, *d* = 0.50 (see Table 5), with individual increases in 14 out of 22 Kichwa infants, *p* = 0.286.

**TABLE 5:** Means and standard deviations for positive affect and gaze at the experimenter for the longitudinal sample.

	Phase 1		Phase 2	
	Münster	Kichwa	Münster	Kichwa
	*M* (SD)	*M* (SD)	*M* (SD)	*M* (SD)
3 months				
Positive affect [0–6]	0.57 (0.56)	0.65 (0.55)	0.71 (0.59)	0.42 (0.32)
Gaze at experimenter [0–1]	0.45 (0.25)	0.60 (0.33)	0.61 (0.28)	0.74 (0.20)
4.5 months				
Positive affect [0–6]	0.38 (0.48)	0.38 (0.34)	0.49 (0.52)	0.64 (0.66)
Gaze at experimenter [0–1]	0.33 (0.28)	0.49 (0.30)	0.39 (0.27)	0.52 (0.27)

*Note: N* = 16 for the Münster and *N* = 22 for the Kichwa sample.

### 3.4 Infants' Gaze at Experimenter at 3 Months

**FIGURE 2:**
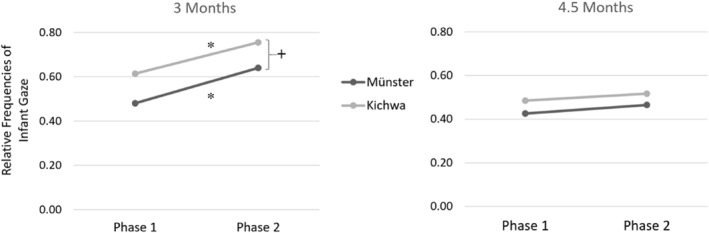
Relative frequencies of gaze at the experimenter during Phase 1 and 2 at 3 and 4.5 months. Scales ranging between 0 and 1. At 3 months, there is a significant increase in attention to the experimenter's face in both cultural milieus and a tendency toward more attention to her face in Kichwa infants across phases. ^+^ *p* < 0.10, *p < 0.05.

With regard to infants' attention to the experimenter's face at 3 months, the effect of phase was significant, *F*(1, 42) = 16.46, *p* < 0.001, *η* ^2^ = 0.28, indicating an increase in attention to the experimenters' face from Phase 1 to Phase 2 across cultures (see Figure 2). On an individual level, there was an increase in 31 out of 44 infants, *p* = 0.010. Additionally, there was a non‐significant main effect of culture with a medium effect size, *F*(1, 42) = 2.97, *p* = 0.092, *η* ^2^ = 0.07. The cultural milieu × phase interaction was not significant, *F*(1, 42) = 0.06, *p* = 0.907, *η* ^2^ = 0.00.

### 3.5 Infants' Gaze at Experimenter at 4.5 Months

Concerning infants' attention to the experimenter's face at 4.5 months, neither the effect of phase *F*(1, 45) = 1.49, *p* = 0.229, *η* ^2^ = 0.03, nor the effect of culture was significant, *F*(1, 45) = 0.43, *p* = 0.513, *η* ^2^ = 0.01 (see Figure 2). Moreover, the cultural milieu × phase interaction was not significant either, *F*(1, 45) = 0.02, *p* = 0.899, *η* ^2^ = 0.00.

### 3.6 Longitudinal Analysis of Infants' Gaze Across Ages

With regard to infants' attention to the experimenter's face, the effect of age was significant, *F*(1, 36) = 14.31, *p* < 0.001, *η* ^2^ = 0.28, indicating that—across phases and cultural milieus—the amount of gaze at the experimenter was generally lower at 4.5 than at 3 months (see Table 5), with individual decrease in 26 out of 38 infants, *p* = 0.023. In addition, the effect of phase was significant, *F*(1, 36) = 8.84, *p* = 0.005, *η* ^2^ = 0.20, indicating a greater attention to the experimenter's face during the high‐intensity stimulation phase than during the medium‐stimulation phase across ages and cultural milieus. On an individual level, 26 out of 38 infants showed this effect, *p* = 0.034. Moreover, the effect of cultural milieu was also significant, *F*(1, 36) = 5.65, *p* = 0.023, *η* ^2^ = 0.14, which highlights that Kichwa infants generally gazed more at the experimenter's face than infants from Münster did.

From all interactions, only the age × phase interaction was significant, *F*(1, 36) = 4.40, *p* = 0.043, *η* ^2^ = 0.11, all other *F*s < 0.22, *p*s > 0.642, *η* ^2^s < 0.01. The significant interaction indicates that changes in infant gaze from Phase 1 to Phase 2 varied between ages. More specifically, according to post‐hoc *t*‐tests, there was a significant increase of infants' gaze from Phase 1 (*M* = 0.54, SD = 0.30) to 2 (*M* = 0.69, SD = 0.24) at 3 months, *t*(37) = −3.83, *p* < 0.001, *d* = 0.62, that was shown by 28 out of 38 infants, *p* = 0.005, but the change in gaze from Phase 1 (*M* = 0.42, SD = 0.29) to 2 (*M* = 0.47, SD = 0.28) was insignificant at 4.5 months, *t*(37) = −1.35, *p* = 0.186, *d* = 0.22. The longitudinal comparison of infant gaze at the experimenter during each phase of the experiment yielded no statistically relevant difference in the amount of gaze at 3 months as compared to at 4.5 months during Phase 1, *t*(37) = 1.77, *p* = 0.085, *d* = 0.29, and a significantly greater amount of gaze at 3 as compared to 4.5 months during Phase 2, *t*(37) = 3.90, *p* < 0.001, *d* = 0.63 (see Table 5). On an individual level, this decrease in gaze across ages was shown by 25 out of 38 infants, *p* = 0.073.

### 3.7 Complementary Analysis of Occurrence of Positive Affect

If one restricts the analysis to the (relative) *occurrence* of positive affective expressions (i.e., proportion of codable intervals with any positive affect), the effects are identical to the cross‐sectional and longitudinal ANOVAs reported here, with specific effects being even more accentuated (see Tables A1 and A2).

## 4 Discussion

In the present cross‐cultural study, we investigated the positive reactivity and focus of attention of 3‐ and 4.5‐month‐old infants during a joyful stimulation standardized face‐to‐face interaction with an experimenter in two cultural milieus that differ in their ethnotheories about infants' ideal affect (Wefers et al. 2022).

At 3 months of age, the interaction effect with a medium effect size indicated that infants from the two cultural milieus tended to respond differently to the increase in positive stimulation from Phase 1 to Phase 2. At the same time, Kichwa infants generally showed a tendency to gaze at the interactive partner's face for longer than infants from Münster, speaking to their heightened interest in an interactional style that did not resonate with their everyday experiences (see e.g., Wefers et al. 2023). Taking both infants' positive reactivity *and* gaze into account, one interpretation is that Kichwa infants engaged in this joyful affect dynamic to some amount during Phase 1, because—as is biologically grounded—the threshold to engage in positively tuned face‐to‐face interaction is very low at 3 months. However, when the experimenter increased stimulating behavior in the second phase, namely by marking, provoking and mirroring infant smiling, the Kichwa dyad did not enter into the dynamic of bidirectional cycling and mutual amplification of positive affect (as described, e.g., by Lavelli and Fogel 2005), because the pattern of stimulation offered by the experimenter was in stark contrast to their previously internalized interactional histories; those were reported to be more centered on bodily proximity to the mother and around low‐arousal positive affect when face‐to‐face interaction is established (Wefers et al. 2022). Moreover, while previous studies pointed to the co‐occurrence of mutual gaze and infant positive affect (Kärtner et al. 2022; Lavelli and Fogel 2002), the gazing behavior of 3‐month‐old Kichwa infants suggests that gaze functions as a *necessary* but not *sufficient* condition for the display of positive affect: that is, high levels of attention to the experimenter's facial expression do not guarantee positive affect in Kichwa infants. It may be that infants engage in positively tuned interactional dynamics during mutual gaze episodes only on the basis of corresponding internalized interactions.

In accordance with our hypothesis, 3‐month‐old infants from Münster reacted with significantly higher average intensities of positive affect than Kichwa infants to the high‐intensity smiles of their interactive partner in the second phase, a finding that is in line with previous naturalistic cross‐cultural studies on the emergence of infant smiling (Kärtner et al. 2022; Wörmann et al. 2012, 2014). Importantly, earlier studies analyzed differences in infant smiling within culturally informed and naturalistic mother‐infant interaction, which makes it impossible to disentangle situational effects due to differences in maternal stimulation from differences in the development of infants' positive affect. Therefore, the current findings are an important complement to these studies by allowing for a stronger inference that the reported cross‐cultural differences in infants' positive affect are *developmental differences*, as the present findings rely on a standardized way of stimulating positive affect in infants.

The cross‐cultural differences at 3 months may be explained by culture‐specific ethnotheories about ideal emotional states in young infants (Wefers et al. 2022). Whereas mothers in Münster might regard intense positive affect as an ego‐focused emotion, which is a welcomed expression of the uniqueness of their child, Kichwa mothers might evaluate it as exaggerated and transgressive (Keller 2007; Markus and Kitayama 1991; Wefers et al. 2022).

At 4.5 months, however, there was an increase in positive affect along with the increase in stimulation in both Kichwa infants *and* infants from Münster, which opens space for discussion about developmental trajectories of culture‐specific affect display. In particular, the question is why there was a substantial change in the affective response pattern specifically in Kichwa infants between 3 and 4.5 months of age (see significant three‐way interaction for positive affect). The converging evidence from previous naturalistic studies generally suggests that cross‐cultural differences in parental ethnotheories function as implicit organizers during parent‐infant interactions, which—during the sensitive phase around 2 months of age—give rise to culture‐specific developmental trajectories in positive affect. Given the reported differences between the cultural milieus with regard to infants' everyday experiences (distal vs. proximal parenting) and regarding parental ethnotheories about infants' ideal affect (Wefers et al. 2022, 2023), the findings of the present study may indicate that Kichwa infants—whose daily interactions are generally more centered on body proximity with their primary caregiver—have accumulated *sufficient* experiences with joyful affect–eliciting face‐to‐face interactions with caregivers only *after* the third month, such that they can join into this specific interactional dynamic at 4.5 months. That is, only between their infants' ages of 3 and 4.5 months might Kichwa mothers have increasingly provided them with opportunities for positively tuned face‐to‐face interactions (i.e., later onset of this dynamic within the Kichwa group), leading to similar levels of emotional reactivity of Kichwa and Münster infants at 4.5 months. However, this interpretation needs to be further consolidated in future longitudinal naturalistic studies.

Importantly, despite the cross‐cultural similarities in 4.5‐month‐old infants' affective reactions during this experimental procedure, infants' everyday experiences and reactions in naturalistic settings are likely to differ systematically across cultures (e.g., due to culture‐specific ethnotheories and parenting styles) and may lead to significant cross‐cultural differences in what *central* interactional routines around infants' positive affect look like in everyday life (Kärtner 2015; Kärtner et al. 2022).

With regard to the development of infant positive affect and focus of attention between 3 and 4.5 months, infants' gaze at the experimenter decreased from 3 to 4.5 months and, at the same time, there was no significant change in the overall level of positive reactivity across ages (i.e., no significant main effect of age). On the one hand, the lack of an increase in the average intensity and in the occurrence of positive affect in Münster infants is surprising (see e.g., Messinger et al. 1999). At the same time, the lack of an overall age effect might indicate that—by 4.5 months—episodes of joyful communication during everyday face‐to‐face interactions (and analogously standardized interactions) might have become more tightly coupled, shorter, and perhaps more effective (Kaye and Fogel 1980; Nomikou et al. 2016). With regard to the decrease in gaze, it might reflect the ongoing developmental shifts at that age, for example, in infants' physical and motoric abilities (Adolph and Berger 2015; von Hofsten and Rosander 1997), which bring about changes within the parent‐infant system, such as new possibilities to communicate about third objects outside the parent‐infant dyad (Nomikou et al. 2016); these triad interactions may draw attention away from faces to surrounding objects (see also Messinger et al. 2012).

Within the same overall project, we analyzed infants' reaction to the sudden *interruption* of the standardized social interaction and—analogous to the present study—findings of Wefers et al. (2023) pointed to cross‐cultural similarities (e.g., evidence for a still‐face effect at 3 and 4.5 months and in both cultural milieus), but also revealed culture‐specific accentuations (e.g., concerning infants' response to an interruption of proximal interaction patterns during a second paradigm, the so‐called no‐touch paradigm).

Several avenues for future research have the potential to overcome some limitations and shortcomings of the present study: One limitation is that we had to exclude 16 infants, who were fussy or cried/whimpered before or during the assessments and therefore could not (continuously) engage in social interaction with a stranger, which may have affected results. Moreover,—as a result of the larger project's dense assessment plan—rescheduling of the assessments could not always be realized. As a further consequence of the relatively high drop‐out rates, the study includes a risk for type 2 error and may not be able to detect differences due to power limitations. Another limitation is that the experimenters' behavior was not coded. Regarding the further development of the research design, future studies could conduct the same standardized interactions with the mother and with a stranger, which would, for example, allow to consider the effects of a possible cross‐cultural difference in infants' familiarity with strangers. Moreover, complementary to the aggregated analyses of infants' positive reactivity reported here, more nuanced analyses of latencies, lengths and intensities of positive affect episodes may serve as fruitful indicators for culture‐specific pathways. To allow for a precise disentanglement of possible (dynamically interacting) sources of cross‐cultural differences in early emotional development, future studies could consider combining longitudinal behavioral with psychophysiological measures (see e.g., Kärtner et al. 2010). Furthermore, to balance ethnocentric biases in the thematic focus of research questions, future research on emotion socialization during infancy should more openly explore affect socialization in proximal care milieus, such as contingent responses of caregivers via voice and body contact equivalent to affect mirroring and its implications for developmental outcomes such as awareness of feelings. In addition, in cross‐cultural infancy studies, the development and application of coding schemes can best be accomplished within intercultural teams representing the participants' cultural milieus and future studies should take this into account. Lastly, the fact that the predictions of the present study were not preregistered, also represents a limitation.

## 5 Conclusion

The present standardized study investigated the development of positive affect during early infancy in two cultural milieus. It provides evidence for cross‐cultural differences in positive affect at 3 months, namely higher intensities of positive affect in response to intense positive stimulation in Münster infants (urban Germany), a cultural milieu that is associated with a preference for high levels of positive affectivity, as compared to Kichwa infants (rural Ecuador). At 4.5 months of age, when attention is increasingly distributed between faces and objects outside the parent‐infant dyad, infants from both milieus responded with similar levels of positive affect to the interactional style of the experimenter. The present study complements the existing literature, as it points to cultural similarities *and* differences using a joyful stimulation standardized face‐to‐face interaction. Moreover, our findings support a threshold model regarding the early development of positive affect, that is, comparable reactivity given sufficient experience. The findings also suggest that—despite cross‐cultural similarities in developmental outcomes (here, infants' positive reactivity at 4.5 months)—developmental pathways differ between cultures depending on how biological potentials (e.g., infants' attentiveness to faces) interact with culturally informed social interactions across the first months of life.

## Author Contributions

**Helen Wefers:** conceptualization, data curation, formal analysis, investigation, methodology, project administration, writing – original draft. **Nils Schuhmacher:** formal analysis, writing – review and editing. **Joscha Kärtner:** conceptualization, formal analysis, resources, supervision, writing – review and editing.

## Open Practices Statement

The aggregated data and the code are available on the Open Science Framework (OSF; https://osf.io/ravcy/?view_only=c3b09dc1becb494d8a0b5a8340b712b5).

## Endnotes

^1^
Following the baseline, there was an interruption phase, however, infants' reactions to the sudden interruption of social interaction is not subject of this publication, but of Wefers et al. (2023).

## Appendix A Reasons for Excluding Assessments at 3 and 4.5 Months

Assessments were not completed or had to be excluded for different reasons such as (i) infants were fussy or cried before the assessment and were not ready to be separated from the parent and interact with a stranger (3 months: *n*_MS_ = 4, *n*_KI_ = 1; 4.5 months: *n*_MS_ = 2, *n*_KI_ = 1), (ii) assessments were interrupted because infants cried consecutively for more than 10 s (3 months: *n*_MS_ = 0, *n*_KI_ = 2; 4.5 months: *n*_MS_ = 0, *n*_KI_ = 1), (iii) assessments were excluded post‐hoc because the experimenter did not strictly follow the protocol (e.g., length of Phase 1 or 2 < 30 s or more than 90 s [instead of 60 s], *n*_MS_ = 0, *n*_KI_ = 2 at 3 months), (iv) error in data storage (*n*_MS_ = 1, *n*_KI_ = 0 at 4.5 months), and (v) whine/whimper for at least 20% of the Phase 1 or 2 time (3 months: *n*_MS_ = 2, *n*_KI_ = 0; 4.5 months: *n*_MS_ = 3, *n*_KI_ = 0).

## Appendix B Effects of Parity and Gender at 3 and 4.5 Months

With regard to the intensity of positive affect at 3 months of age, neither main effects of gender and parity nor their interactions with culture and phase were significant when they were included separately as additional factors in the ANOVA.

When including gender as an additional factor at 4.5 months of age, the pattern of effects of phase, *F*(1, 43) = 5.30, *p* = 0.026, *η* ^2^ = 0.11, cultural milieu, *F*(1, 43) = 0.31, *p* = 0.583, *η* ^2^ = 0.01, and cultural milieu × phase, *F*(1, 43) = 0.15, *p* = 0.705, *η* ^2^ = 0.00, was identical to the results of the two‐factorial ANOVA reported above. In addition, there was a significant cultural milieu × phase × gender interaction, *F*(1, 43) = 10.31, *p* = 0.003, *η* ^2^ = 0.19, and a marginally significant cultural milieu × gender interaction, *F*(1, 43) = 2.93, *p* = 0.094, *η* ^2^ = 0.06, indicating that the effect of gender on infant affect varied across cultures.

In summary, we found cultural differences in phase × gender interaction effects at 4.5 months: While Kichwa girls (but not boys) showed a significant increase in positive affect from Phase 1 (*M* = 0.38, SD = 0.32) to Phase 2 (*M* = 0.77, SD = 0.70), *t*(16) = −2.97, *p* = 0.009, *d* = 0.54, Münster boys (but not girls) showed a significant increase in positive affect from Phase 1 (*M* = 0.31, SD = 0.49) to Phase 2 (*M* = 0.64, SD = 0.53), *t*(11) = −3.17, *p* = 0.009, *d* = 0.37. Overall, these few exceptions concerning the effect of gender on infant affect at 4.5 months might indicate that—across cultures—gender might play a differential role in the socialization of affect, even in early infancy; future studies should follow up on this.

When including parity as an additional factor at 4.5 months of age, neither the main effect of parity nor its interaction with culture and phase was significant. Regarding infant gaze at 3 and 4.5 months of age, neither the main effects of gender and parity nor their interactions with culture and phase were significant when they were included as additional factors into separate ANOVAs.t

## Appendix C

**TABLE A1:** Descriptive information and effect sizes for the mixed ANOVAs with occurrence of positive affect as dependent variable (cross‐sectional sample).

	Phase 1		Phase 2		Partial *η* ^2^		
	Münster	Kichwa	Münster	Kichwa	CM	P	CM × P
	*M* (SD)	*M* (SD)	*M* (SD)	*M* (SD)			
3 months							
Occurrence of positive affect [0–1]	0.24 (0.20)	0.30 (0.24)	0.33 (0.23)	0.20 (0.15)	0.01	0.00	0.17**
4.5 months							
Occurrence of positive affect [0–1]	0.20 (0.20)	0.17 (0.17)	0.25 (0.19)	0.25 (0.25)	0.00	0.10*	0.01

*Note:* Occurrence of positive affect at 3 months: *N* = 20 for the Münster sample and *N* = 24 for the Kichwa sample. At 4.5 months: *N* = 20 for the Münster sample and *N* = 27 for the Kichwa sample. At 3 months, post‐hoc *t*‐tests yielded a non‐significant decrease of the occurrence of infant positive affect from Phase 1 to Phase 2 in the Kichwa sample with a small to medium effect size, *t*(23) = 2.06, *p* = 0.051, *d* = 0.42, and a statistically significant increase across phases in Münster, *t*(19) = −2.11, *p* = 0.048, *d* = 0.47. Comparing the two cultures directly, post‐hoc *t*‐tests indicated a significant difference in the occurrence of positive affect during Phase 2, *t*(42) = −2.29, *p* = 0.027, *d* = 0.67.

Abbreviations: CM = cultural milieu; P = phase.

**p* < 0.05, ***p* < 0.01; two‐tailed.

## Appendix D

**TABLE A2:** Descriptive information for the mixed ANOVAs with occurrence of positive affect as dependent variable (longitudinal sample).

	Phase 1		Phase 2	
	Münster	Kichwa	Münster	Kichwa
	*M* (SD)	*M* (SD)	*M* (SD)	*M* (SD)
3 months				
Occurrence of positive affect [0–1]	0.24 (0.20)	0.32 (0.24)	0.36 (0.25)	0.21 (0.14)
4.5 months				
Occurrence of positive affect [0–1]	0.19 (0.21)	0.20 (0.17)	0.24 (0.21)	0.29 (0.26)

*Note: N* = 16 for the Münster and *N* = 22 for the Kichwa sample. From all main effects and interactions, only the age × phase × cultural milieu interaction was significant, *F*(1, 36) = 7.55, *p* = 0.009, *η* ^2^ = 0.17**, all other *F*s < 2.56, *p*s > 0.117, *η* ^2^s < 0.07. To analyze this further, we conducted post‐hoc ANOVAs separately for each cultural milieu with age (3 vs. 4.5 months) and phase (1 vs. 2) as within‐subject factors: With regard to age‐related changes in occurrence of positive affect in Kichwa infants, there was a significant age × phase interaction, *F*(1, 21) = 7.20, *p* = 0.014, *η* ^2^ *=* 0.26. More precisely, according to post‐hoc *t*‐tests, whereas the non‐significant *decrease* in occurrence of positive affect from Phase 1 to Phase 2 of 3‐month‐old Kichwa infants had a medium effect size, *t*(21) = 2.08, *p* = 0.050, *d* = 0.54, 4.5‐month‐old Kichwa infants significantly *increased* in positive affect from Phase 1 to Phase 2, *t*(21) = −2.23, *p* = 0.037, *d* = −0.45; two‐tailed.
